# Conglomeration of Trabecular and Psammomatoid variants of juvenile ossifying fibroma – a rare case report

**DOI:** 10.1002/ccr3.910

**Published:** 2017-04-17

**Authors:** Lipsa Bhuyan, Abikshyeet Panda, Kailash Chandra Dash, Mohiddin S. Gouse, Kiran Misra

**Affiliations:** ^1^ Department of Oral and Maxillofacial PathologyKalinga Institute of Dental SciencesKIITBhubaneswarOdishaIndia

**Keywords:** Cone beam computed tomography, juvenile ossifying fibroma, psammomatoid, trabecular

## Abstract

Juvenile ossifying fibroma is an uncommon benign fibro‐osseous lesion occurring in the craniofacial skeleton with a high recurrence rate. It has two distinct histopathologic variants: one trabecular and the other which are exclusive to each other. This case reveals a rare and unique combination of both the patterns in the same lesion.

## Introduction

Fibro‐osseous lesions [FOL] affecting the jaws and the craniofacial bones are diverse processes in which the normal architecture of bone is replaced by fibrous tissue containing varying degree of mineralization 1. Juvenile ossifying fibroma [JOF] is a rare benign locally aggressive tumor with high propensity to recur. It is believed to cause considerable diagnostic challenges due to its rapid growth and osteolytic nature 2. Two histologic patterns were identified by El‐Mofty in the year 2002 and categorized JOF into psammomatoid JOF [PsJOF] and trabecular JOF [TrJOF] 3. After a thorough search in the databases of PubMed, Medline, and Google scholar, only one case with combined histopathologic patterns was reported in the skull base 4. We report a rare case of JOF with both the histopathologic patterns conglomerated in the same lesion located in the maxilla.

## Case Report

**Figure 1:**
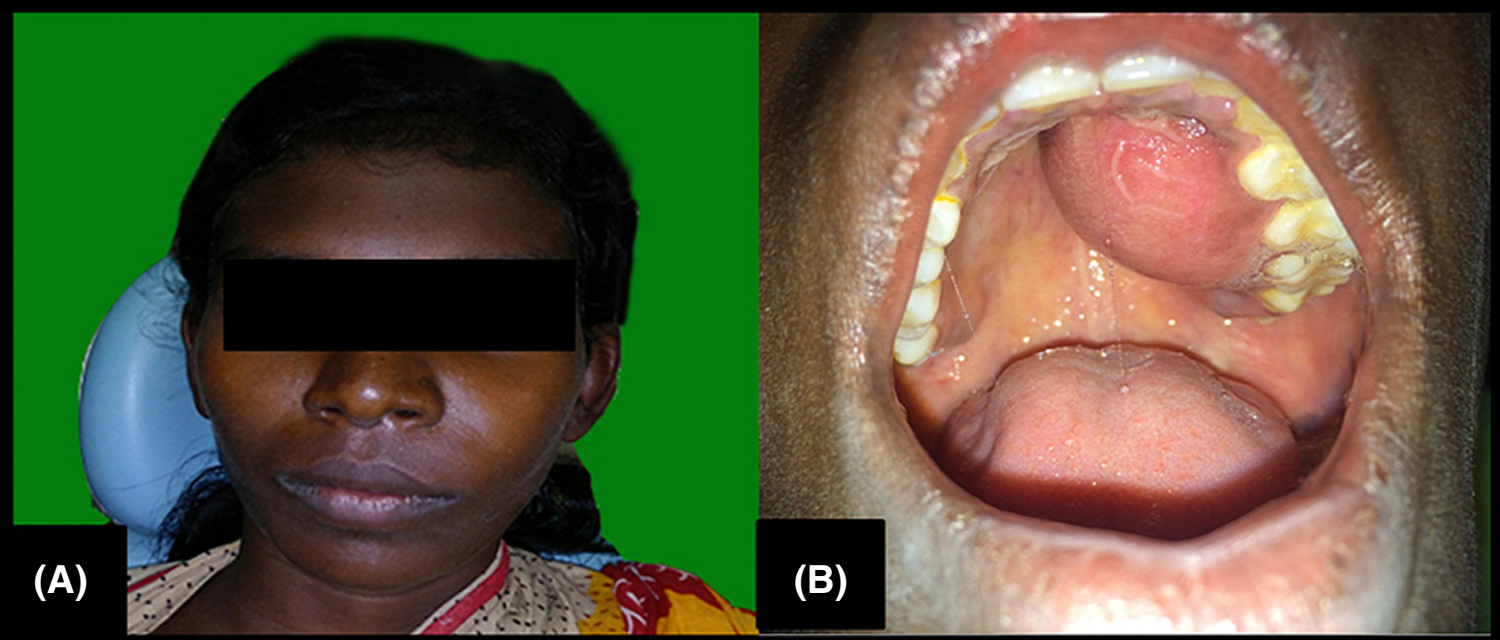
(A) Diffuse expansile extra‐oral swelling involving left maxilla with facial asymmetry. (B) Well‐defined intra‐oral swelling involving the left half of the palate crossing the midline.

A 20‐year‐old female presented to the department of Oral Pathology and Microbiology, Kalinga Institute of Dental Sciences, with a rapidly progressive painless swelling on left side of the face of one and a half months duration. She had no complaint of pain, visual disturbances, dysphagia, or dyspnea. Her past medical history was noncontributory. Extra‐oral examination revealed a diffuse, bony hard, nontender expansile swelling involving left maxilla measuring about 5 cm by 4 cm in its greatest diameter causing significant facial asymmetry. Intra‐orally, a well‐defined solitary swelling involving the left half of the palate measuring about 3 cm by 2 cm was noted (Fig. 1A and B). The lesion extended from 24 to 26 with little displacement of the associated second premolar. On palpation, the swelling was hard and nontender.

Hematological findings were unremarkable, and serum alkaline phosphatase levels were slightly elevated (125 U/L).

**Figure 2:**
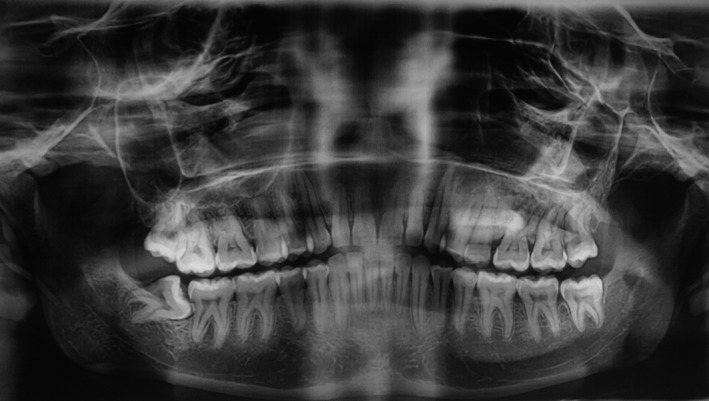
Orthopantomogram showing mixed radiolucent and radiopaque mass with diffuse border in the left maxilla.

**Figure 3:**
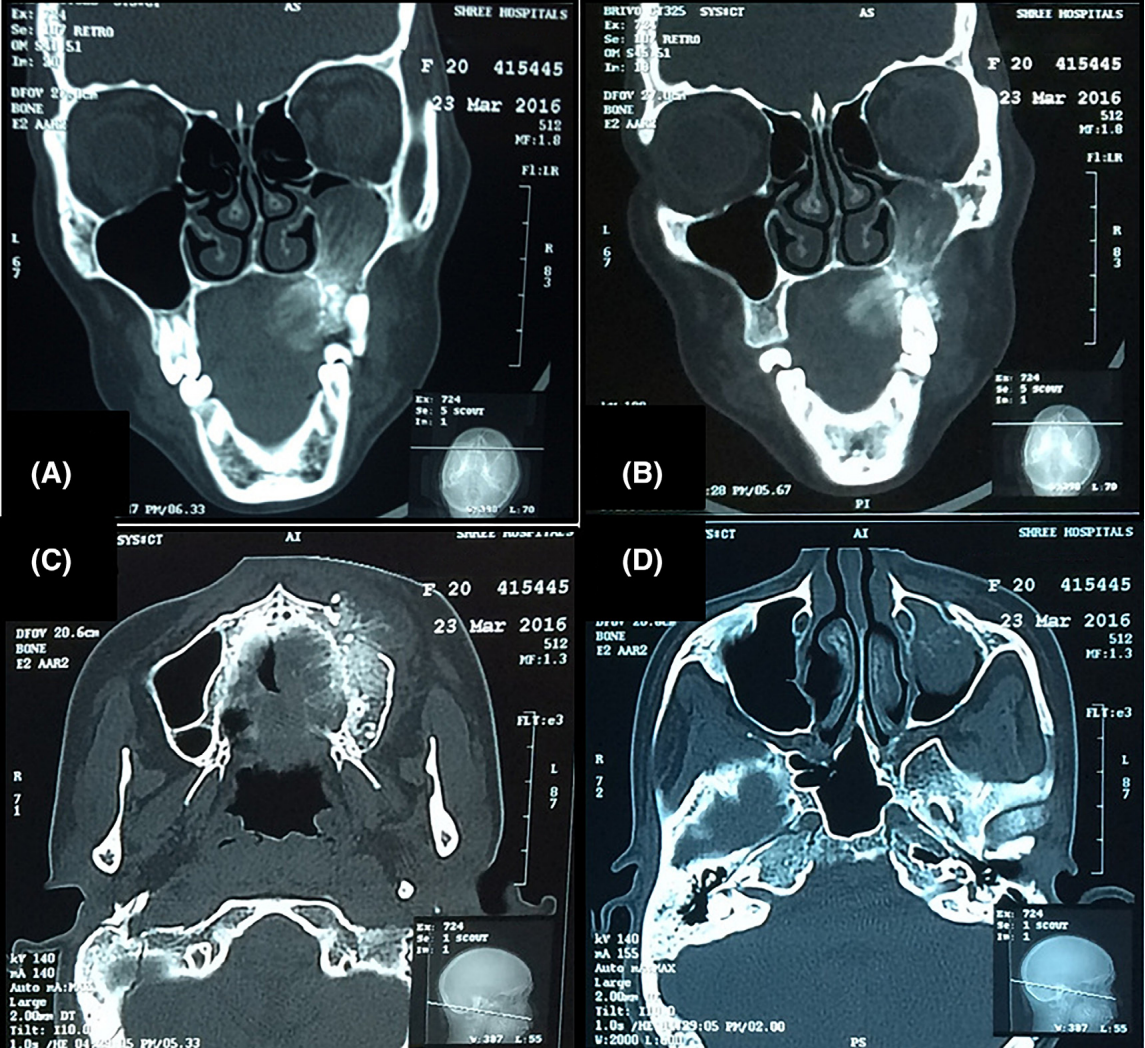
(A and B) Coronal CT scan showing hyperdense mid‐facial mass involving the left maxillary sinus. (C and D) Axial CT scan view showing mixed‐density mass with expansion of the left palate with diffuse borders.

Radiological investigations included orthopantomogram (OPG), computed tomography (CT), and cone beam computed tomography (CBCT) scan. OPG revealed mixed radiolucent and radiopaque lesion obliterating the left maxillary sinus giving a ground glass appearance (Fig. 2). Axial and coronal CT scan showed hyperdense mid‐facial mass involving the left maxillary sinus and mixed‐density mass with expansion of the left palate with diffuse borders (Fig. 3A and B).

**Figure 4:**
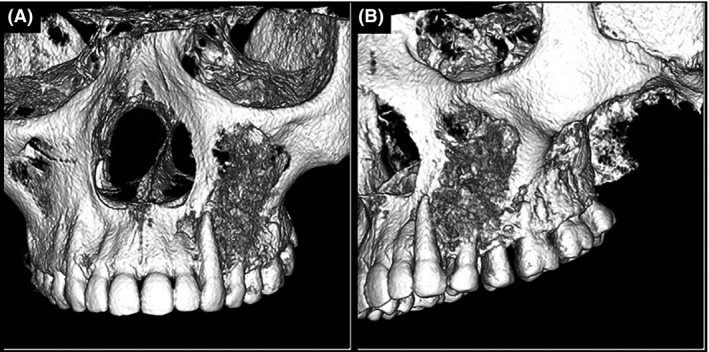
(A and B) CBCT 3D reconstruction of the maxilla showing buccal cortical plate destruction with numerous spicules of calcifications on the left side.

Cone beam computed tomography from the axial, coronal, and sagittal planes showed a lesion of mixed density with ill‐defined borders involving the maxilla and extending into the palate. 3D reconstruction of the maxilla showed numerous spicules of calcifications and slight displacement of the first and second premolar (Fig. 4A and B).

**Figure 5:**
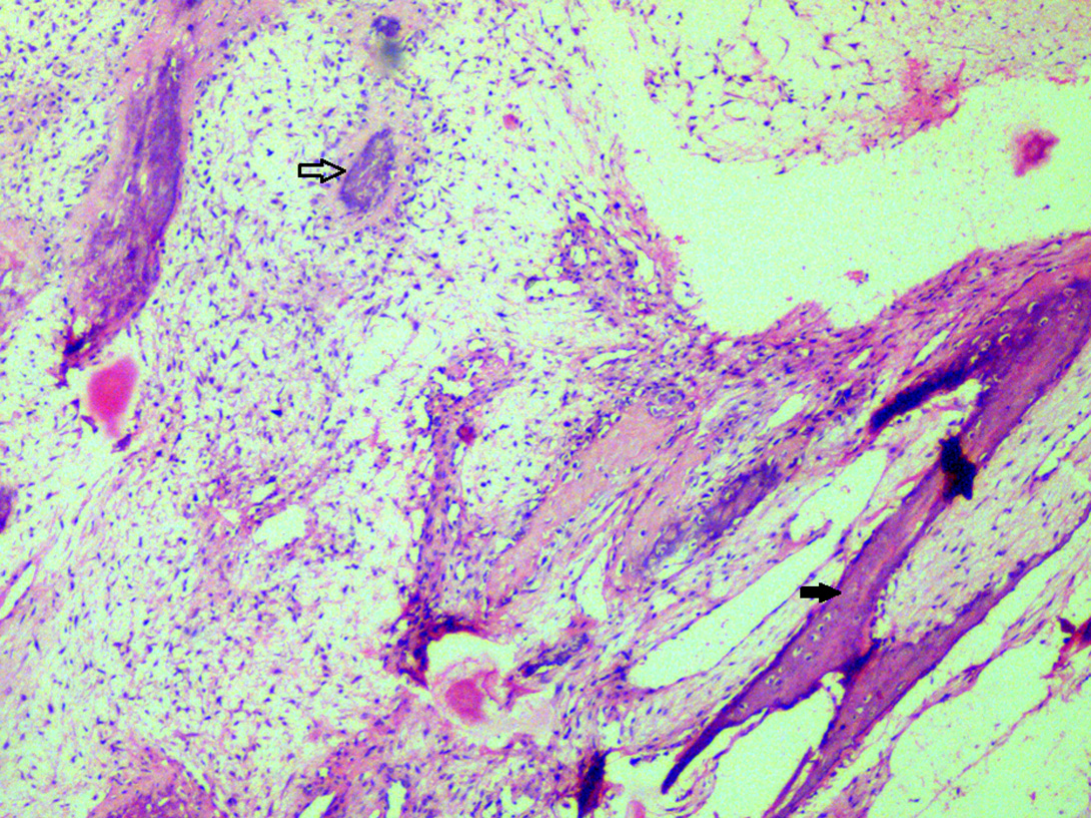
Hematoxylin and eosin‐stained sections demonstrating a highly cellular mesenchymal stroma with bony trabeculae (solid arrow) and psammomatoid structures (hollow arrow) (40 X).

**Figure 6:**
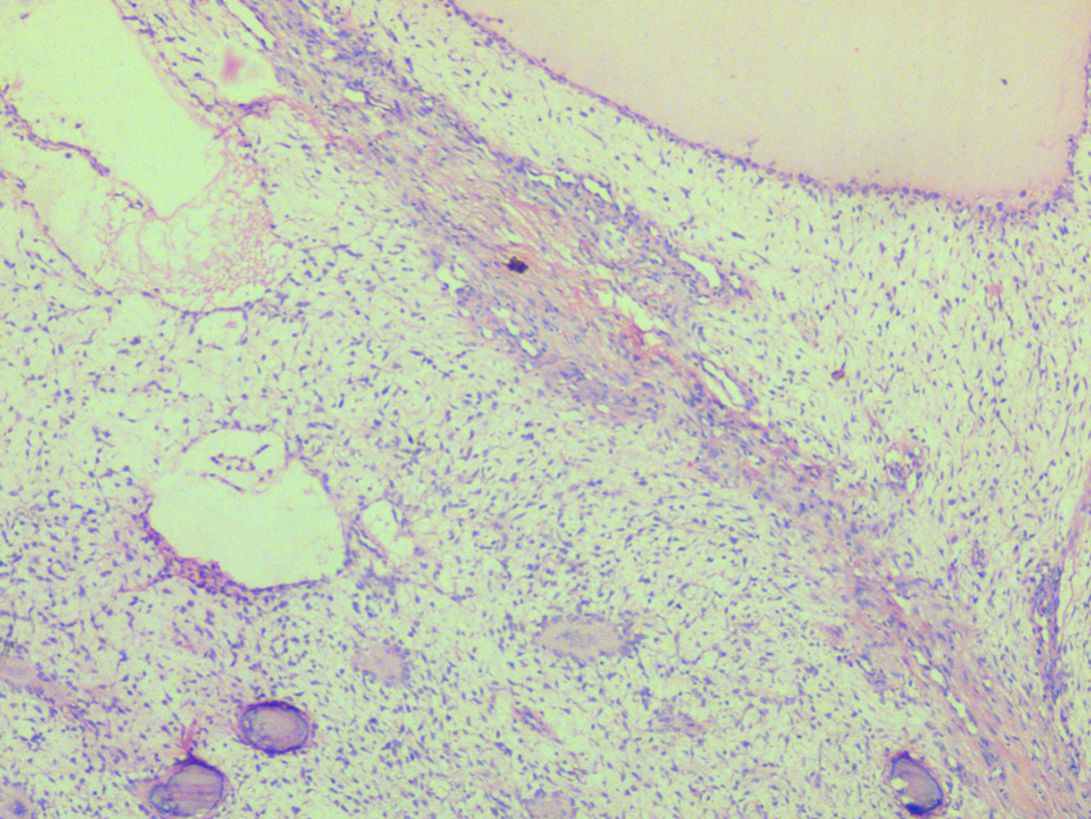
Mesenchymal stroma showing myxoid and fibrous area with cystic degeneration (100 X).

**Figure 7:**
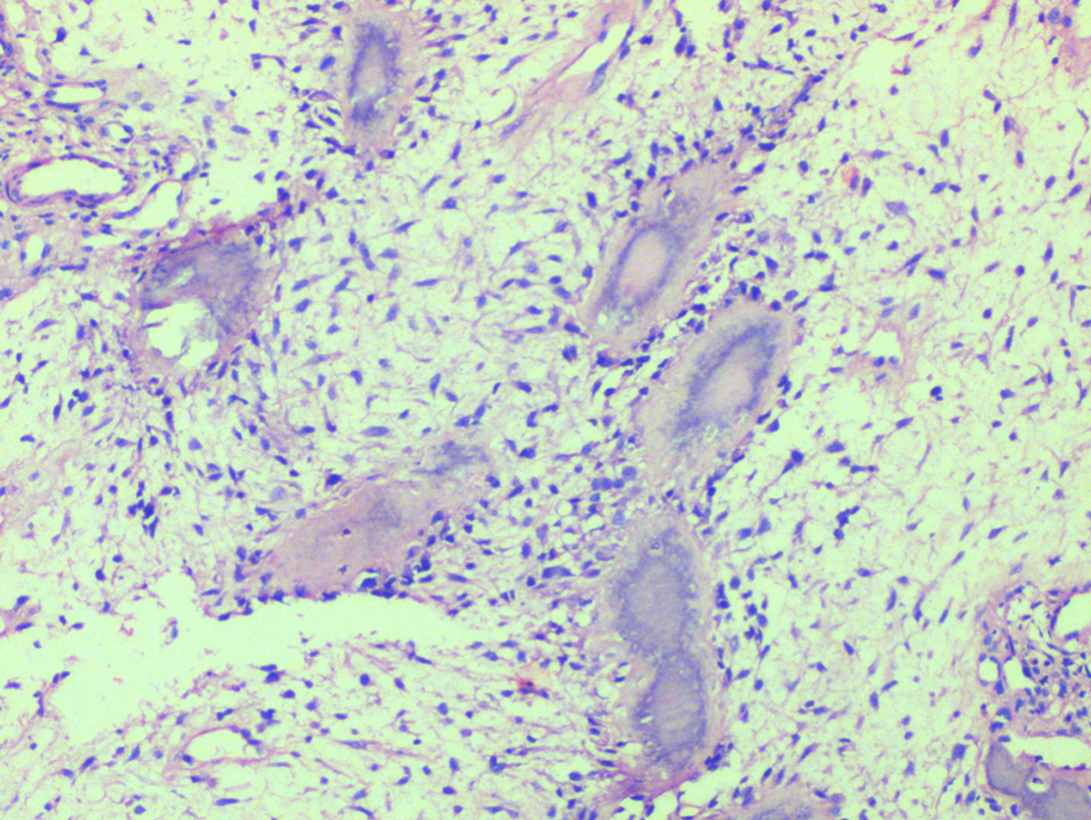
The psammomatoid ossicles showing deeply basophilic calcification with brush border appearance surrounded by an eosinophilic area (400 X).

Pre‐operative incisional biopsies were performed. Gross examination of the specimen showed multiple grayish white mass which were firm on palpation. Microscopic examination of the hematoxylin and eosin‐stained sections demonstrated bony trabeculae of varying size along with numerous psammomatoid structures (Fig. 5). Plump spindle‐shaped fibroblasts were scattered in a loose connective tissue stroma with myxoid changes in most of the areas. The psammomatoid ossicles showed deeply basophilic calcification with brush border appearance in eosinophilic background. Trabeculae consisted predominantly of woven bone with osteoblastic rimming. Cystic degeneration was seen in few areas (Figs 6 and 7). Surgical excision of the lesion using left buccal vestibular incision was performed. A clear surgical bed was achieved. Excisional biopsy confirmed the same. The patient was recalled for follow‐up on a regular interval of 6 months.

## Discussion

Fibro‐osseous lesions are a diverse group of lesions affecting the craniofacial bones and jaws characterized by the replacement of normal bone architecture by fibrocollagenous tissue containing foci of mineralization of varying degrees which may be osseous or cementum‐like in appearance 5. Johnson first used the term “juvenile ossifying fibroma” in 1952, while describing aggressive variants of ossifying fibroma affecting the craniofacial bones of children. It is a relatively uncommon FOL and is well demarcated from other FOLs based on the age, site, clinical behavior, and histopathologic features 3.

Juvenile ossifying fibroma has a higher incidence in children and young adults, although it can also occur at an older age. According to classification by El‐Mofty, the average age of incidence in trabecular JOF is 8½–12 years, whereas its 16–33 years for psammomatoid 3. Slootweg et al. categorized JOF based on the age of occurrence into JOF‐WHO with mean age of occurrence 11.8 years and JOF‐PO (psammoma‐like ossicles) with mean age of occurrence 22.6 years 5. Patient's age in our case was 20 years.

Due to its aggressive nature and high tendency to recur compared to other FOL, it is also named as aggressive ossifying fibroma 4. In our case, the patient gave a history of rapidly growing mass which had noticeably increased in size within a short span of 6 weeks. This emphasizes the aggressive behavior of the lesion. Although mobility was not present, displacement of the associated teeth (24, 25) termed “dental anarchy” by Osunde et al. was seen 2.

There was a slight male predilection in demographics of series of cases of PsJOF by Johnson et al. 6, Makek 7, Slootweg et al. 8, and El‐Mofty 3, whereas female predilection was reported by Dehner 9. In case of TrJOF, male predilection was reported by Makek 6, Slootweg et al. 5, and El‐Mofty 3, whereas female predilection was reported by Dehner 9 and Slootweg and Muller 10.

Because of limited number of cases in the literature, the incidence of JOF is still unknown. PsJOF is more frequently reported than TrJOF. In a comprehensive review of the literature, the number of cases reported for PsJOF is four times more than TrJOF 3. Only one case with co‐existence of both trabecular and psammomatoid pattern located in the skull base was reported by Bohn OL et al. 5. We herein report a similar case in the maxilla.

Microscopically, TrJOF is characterized by osteoid matrix incorporating plump eosinophilic osteoblastic cells. Progressive calcification of immature cellular osteoid may form anastomosing trabeculae which is not clearly distinguishable from the surrounding fibroblastic stroma resembling paintbrush strokes 3. Mature lamellar bone is not seen. Cystic degeneration, aggregates of multinucleated giant cells and mitotic activity of the stromal cells, may be seen 4. Monostotic fibrous dysplasia poses a prominent differential diagnosis. Its slow growing nature which tends to stabilize on skeletal maturity, ill‐defined margins, peculiar Chinese letter pattern of mature lamellar bony trabeculae without osteoblastic rimming, and less cellular stroma differentiates it from TrJOF. Aneurysmal bone cyst, central giant cell granuloma, Burkitt lymphoma, and other malignant bone tumors like osteoblastoma and osteosarcoma can be differentiated by histopathology 2, 11.

“Psammoma” is derived from a Greek word “psammos” which means “sand.” Sometimes calcification occurs in concentric lamellated configuration resembling grains of sand called as psammoma bodies as seen in papillary thyroid carcinoma 12. The true psammoma bodies in extracranial meningioma are acellular spherical entities which are haphazardly distributed, lack‐associated osteoclasts, and osteoblastic rimming and are positive for EMA and vimentin 4. The unique spherical bodies of JOF were termed “psammoma‐like bodies” by Gogl in 1949. Microscopically, they are acellular mineralized ossicles or may show few scattered cells. They usually show irregular thread like or thorn‐like calcified strands giving it a brush border appearance in a hyalinized background. The ossicles may also have a deeply basophilic concentrically lamellated calcification in a loose fibroblastic to intensely cellular stroma. Ultrastructural studies of the psammoma‐like bodies show small spicules and needle‐like crystalloids at periphery radiating from a dark rim of crystals 13.

The resemblance of psammoma‐like ossicles to cementicles in central cemento‐ossifying fibroma (CCOF) often creates a diagnostic confusion. Few authors argued it to be the same entity. JOF is a well‐defined clinical and histological entity. The CCOF is well encapsulated and can be easily enucleated, whereas JOF is not capsulated and shows diffuse or radio‐opaque border with destruction of adjacent structures 14. Sometimes cortical thinning and perforation are also seen. CCOF has its origin from periodontal ligament, and therefore, they are usually present in the tooth‐bearing areas of the jaw predominantly in the mandible. PsJOF mostly occurs in orbital bones and paranasal sinuses, that is, ethmoid and maxillary sinus 4. Histologically, the cementum‐like particles are basophilic, nonuniform in size, have a random distribution and may coalesce, whereas psammomatoid bodies show a concentrically lamellated basophilic center with eosinophilic fringe, uniform in size, and distribution. The PsJOF sometimes show mild myxoid change and microcystic spaces in the fibrocelluar stroma 3, 15.

Maldevelopment of basal generative mechanism which is prerequisite for root formation and the presence of translocation by nonrandom chromosomal breakpoints at Xq26 and 2q33 have been reported to be the possible pathogenesis of JOF 16, 17.

Although malignant transformation is very rare, high recurrence rate of 30–56% in both patterns of JOF is seen 3. However, it has been reported to be associated with secondary changes such as aneurysmal bone cysts and hemorrhage 9. Due to its aggressive nature, surgical resection rather than conservative curettage is recommended. Radiotherapy may lead to malignant transformation (0.4% to 40%) and may have potential harmful late effects in children. Because of its propensity to recur, immediate reconstruction is avoided followed by a long clinical and radiological follow‐up 15, 18. In this case too, the mass was surgically excised with a clear surgical bed. Reconstruction was planned after 2 years of recurrence‐free period. She is now under close follow‐up. She will be clinically observed and radiologically assessed in an interval of 6 months.

## Conclusion

To the best of our knowledge, the occurrence of trabecular and psammomatoid pattern co‐existing in a single tumor mass of JOF is a rare entity, with only one previously reported case located in the skull base. Although various studies have claimed that there are two distinct patterns of JOF, their coexistence may be due to cumulative effect of their pathogenesis or may be two lesions in collision. Further studies should be carried out on the nature and pathogenesis of such lesions. Moreover, our case is probably the first reported‐case with combination of both the entities of JOF located in maxilla. Diagnosis of these lesions requires meticulous clinical, radiologic, and histopathologic correlation. The present case represents an unusual presentation of JOF in terms of its aggressive behavior and rare histopathologic presentation, thus emphasizing its early diagnosis, management, and long‐term follow‐up.

## Authorship

LB: stuctured the case report and the concept. AP: Structured the manuscript. KCD : involved in the case report description. MSG: involved in the discussion. KM: involved in the clinical Case history, follow‐up, and discussion.

## Conflict of Interest

None declared.
